# Multi-step ahead thermal warning network for energy storage system based on the core temperature detection

**DOI:** 10.1038/s41598-021-93801-9

**Published:** 2021-07-28

**Authors:** Marui Li, Chaoyu Dong, Xiaodan Yu, Qian Xiao, Hongjie Jia

**Affiliations:** 1 Key Laboratory of Smart Grid of Ministry of Education, Tianjin University, Tianjin, 300072 China; 2 Key Laboratory of Smart Energy & Information Technology of Tianjin Municipality, Tianjin, 300072 China; 3 Department of Electrical and Electronic Engineering, Imperial College London, London, SW7 2AZ UK

**Keywords:** Electrical and electronic engineering, Energy storage

## Abstract

The energy storage system is an important part of the energy system. Lithium-ion batteries have been widely used in energy storage systems because of their high energy density and long life. However, the temperature is still the key factor hindering the further development of lithium-ion battery energy storage systems. Both low temperature and high temperature will reduce the life and safety of lithium-ion batteries. In actual operation, the core temperature and the surface temperature of the lithium-ion battery energy storage system may have a large temperature difference. However, only the surface temperature of the lithium-ion battery energy storage system can be easily measured. The estimation method of the core temperature, which can better reflect the operation condition of the lithium-ion battery energy storage system, has not been commercialized. To secure the thermal safety of the energy storage system, a multi-step ahead thermal warning network for the energy storage system based on the core temperature detection is developed in this paper. The thermal warning network utilizes the measurement difference and an integrated long and short-term memory network to process the input time series. This thermal early warning network takes the core temperature of the energy storage system as the judgment criterion of early warning and can provide a warning signal in multi-step in advance. This detection network can use real-time measurement to predict whether the core temperature of the lithium-ion battery energy storage system will reach a critical value in the following time window. And the output of the established warning network model directly determines whether or not an early emergency signal should be sent out. In the end, the accuracy and effectiveness of the model are verified by numerous testing.

## Introduction

**Figure 1:**
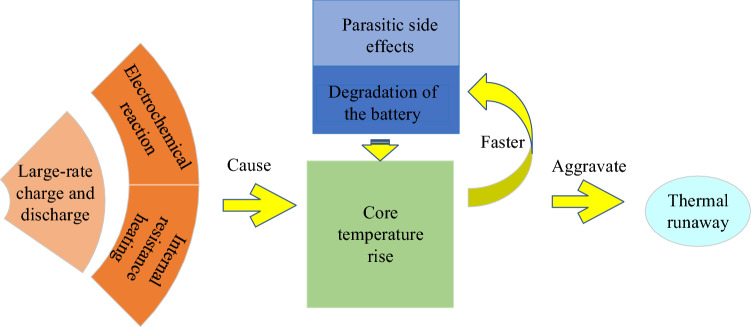
The cause and influence of the rise of core temperature.

Due to environmental pollution, climate change, and the depletion of non-renewable resources, fossil energy is gradually replaced by clean electricity. As an important part of the energy system, the energy storage system of batteries is widely used because of the need for fast response to energy demand and the improvement of battery performance. Lithium-ion batteries are more widely used in the energy storage system than other types of batteries because of their high energy density, long life, low self-discharge rate, and environmental protection^1^. However, temperature-related problems, such as the shortening of cycle life at high temperature and poor performance at low temperature, still hinder the wide application of lithium-ion battery energy storage system in the expanding energy storage market^2^. The decrease of ionic conductivity at low temperature will lead to large voltage polarization and slow solid-state diffusion, which may lead to lithium plating at the anode, thus reducing the life and safety of lithium-ion batteries^3^. The high operating temperature will lead to faster parasitic side effects and significantly accelerate the degradation of the battery^4^. Generally, in the actual use of lithium battery energy storage system, the situation of high temperature is significantly higher than that of low temperature. This is because a lot of heat will be generated in the lithium-ion battery energy storage system due to the electrochemical reaction and internal resistance heating during the charging and discharging process, and the heat generated will cause the temperature of the energy storage system to rise. In addition, the heat transfer will lead to the uneven temperature distribution. So the cycle life and performance of batteries will be greatly affected. In large battery modules and batteries, the accumulation of heat and temperature rise will accelerate the exothermic reaction, and even lead to thermal runaway in severe cases^5^. The cause and influence of the rise of core temperature are shown in Fig. 1. So it is very important to monitor and predict the temperature of the energy storage system.

Due to the heat generation and heat dissipation inside the lithium battery energy storage system, there may be a large temperature difference between the surface temperature and the core temperature of the lithium battery energy storage system^6^. When the heating of the battery is large, the core temperature of the energy storage system will be significantly higher than the surface temperature, and the core temperature of the energy storage system will first reach the critical point. Therefore, in the design of the energy storage system thermal management system, if only the surface temperature is used to determine the safety level of the energy storage system, the energy storage system may be in a dangerous state. However, usually, only the surface temperature of the lithium battery energy storage system can be measured in real-time. As one of the key parameters of thermal state estimation, core temperature is difficult to measure directly^7^. Although there have been studies to measure the internal temperature of the battery in situ by integrating the temperature sensor^8^, it has not been commercialized due to the increased cost and uncertain potential threat. So it is necessary to predict and estimate the core temperature of the lithium battery energy storage system.

At present, the research of lithium-ion batteries’ core temperature estimation is mainly divided into mathematical analysis method based on model and data-driven method based on data. Researches established different models to estimate the temperature distribution of lithium-ion batteries^9–15^. Although they can accurately estimate the real temperature distribution of lithium-ion batteries, it is difficult to use them for real-time estimation due to a large number of equations and parameters. To reduce the computational complexity and complexity of the model, researchers proposed the node methods to estimate the temperature of several key nodes of lithium-ion batteries^16–19^, which can accurately estimate the core temperature of the lithium-ion battery, and have been widely used. However, it may be time-consuming to obtain the heating parameters in the model^2^. In addition, the researchers proposed a new non-invasive measurement of the core temperature of the lithium-ion cell^20,21^. This method does not need to build a parameter-based model, but rather solves the relationship between the core temperature and outside surface temperature distribution as functions of time. But these methods all need some parameter information of battery and some calculation.

Deep learning has been widely used in various fields, because even novices are easy to carry out end-to-end learning without human intervention^22^. If there are enough reliable data, an excellent prediction network can be trained. Long and short-term memory neural network (LSTM) is an excellent recurrent neural network, and has been widely used in safety monitoring. Li et al. combined the long-term memory neural network with the equivalent circuit model of lithium battery by adaptive enhancement method and proposed a fault diagnosis method based on voltage prediction^23^. Wang et al. proposed a power prediction and anomaly detection method based on LSTM neural network, and its advantages are verified by real experiments^24^. In order to avoid the complex process of model building and parameter identification, many researchers have used data-driven methods based on a large number of real data and deep learning to predict the temperature of lithium-ion batteries^2,25,26^. However, most of these studies only predict temperature, seldom predict multi-step temperature changes and give early warning.

The main work of this paper is as follows. Firstly, this paper introduces the method of estimating the core temperature using the two-node equivalent thermal network model of the battery, and points out the shortcomings of the current work. Then, combining multi-step temperature prediction and thermal warning, a multi-step ahead thermal warning network for lithium-ion battery energy storage system is established to judge whether the temperature is out of bounds in multiple future steps. The multi-step ahead thermal warning network is an integrated model of two long and short-term memory neural networks. Two long and short-term memory neural networks are used to train the slow and fast characteristics related to the core temperature changes of the energy storage system. And the neural network established in this paper is a classification network. The model is then trained with a large amount of data. The input of the model is measurable data, and the surface temperature difference between two sampling points is introduced. And the output of the model is 0–1 signals representing whether the core temperature is abnormal at the next few sampling points. In this paper, the ability of core temperature early warning is added to the trained model. Unlike the simple method of judging whether or not the predicted temperature is out of bounds, the multi-step thermal warning network established in this paper directly implements the function of early warning for core temperature anomalies of the energy storage system, which reduces the judgment operation and improves the safety of energy storage system.

## Battery core temperature

### Equivalent thermal network model

**Figure 2:**
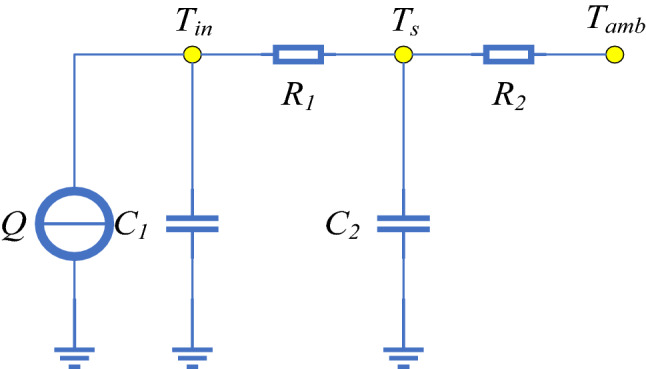
Two-node thermal model.

The battery equivalent thermal network model is shown in Fig. 2^27,28^. Here, *Q* is the heat generation rate of lithium-ion batteries, *R*_1_ and *R*_2_ denote the thermal resistances between the inside and the surface of the battery and between the surface and the environment, respectively, and *C*_1_ and *C*_2_ are the corresponding internal and surface heat capacity of the battery. Finally, *T*_*in*_, *T*_*s*_, and *T*_*amb*_ denote the core, the surface temperature of the battery, and the ambient temperature of the environment, respectively. The mathematical expression of heat transfer is as follows^29,30^:

1 $$K_{1} = 1/R_{1} ,\quad K_{2} = 1/R_{2}$$

2 $$C_{1} *\frac{{dT_{{in}} }}{{dt}} = Q - K_{1} *(T_{{in}} - T_{s} )$$

3 $$C_{2} \frac{{dT_{s} }}{{dt}} = K_{1} *(T_{{in}} - T_{s} ) - K_{2} *(T_{s} - T_{{amb}} )$$

Under a large load current, the heat generation of the battery arises mainly from the ohmic heat^17^, which is proportional to the internal resistance of the battery by

4 $$Q = R*i^{2}$$

*i* is the battery terminal current, *R* denotes the internal resistance of the battery. Since the internal resistance of the battery does not vary greatly with SOC, as long as the battery in a limited region ranging from 20 to 80% of the SOC, *R* is assumed to be a function of the battery’s core temperature *T*_*in*_ only^17^.

### Parameter identification of equivalent thermal network model

In this section, we illustrate a general procedure for identifying the input, output, and parameters of the model. Applying the following discretization.

5 $$\frac{{dT(k)}}{{dt}} = \frac{{z - 1}}{t}*T(k)$$

6 $$zT(k) = T(k + 1)$$

and setting Δt = 1, Eqs. (2) and (3) reduce to

7 $$T_{{in}} (k) - T_{{in}} (k - 1) = a_{1} Q(k - 1) + a_{2} (T_{s} (k - 1) - T_{{in}} (k - 1))$$

8 $$T_{s} (k) - T_{s} (k - 1) = b_{1} (T_{{in}} (k - 1) - T_{s} (k - 1)) + b_{2} (T_{{amb}} (k - 1) - T_{s} (k - 1))$$

where

9 $$a_{1} = \frac{1}{{C_{1} }},~\quad a_{2} = \frac{{K_{1} }}{{C_{1} }},\quad ~b_{1} = \frac{{K_{1} }}{{C_{2} }},\quad b_{2} = \frac{{K_{2} }}{{C_{2} }}$$

Here, Eq. (7) describes that the change of core temperature of the battery is related to heat production and the temperature difference between core temperature and surface temperature; while Eq. (8) entails that the change of the surface temperature of the battery is related to not only the difference between the core temperature and the surface temperature, but also the difference between the surface temperature and the ambient temperature. Since Eq. (7) is decoupled from Eq. (8), coefficients *a*_1_ and *a*_2_ are first estimated using the forgetting factor recursive least squares method in the first stage, followed by an estimation of *b*_1_ and *b*_2_ in the second. As soon as coefficients *a*_1_, *a*_2_, *b*_1_, and *b*_2_ are determined, model parameters *K*_1_*, K*_2_*, C*_1_, *C*_2_ are obtained by solving Eq. (9).

As an effective approach for system identification, the forgetting factor recursive least squares (FFRLS) algorithm identifies the parameters by minimizing the squares of the generalized errors. FFRLS formulas are deduced as

10 $$\left\{ {\begin{array}{*{20}l} {\hat{\theta }(k) = \hat{\theta }(k - 1) + K(k)\left[ {y(k) - \varphi ^{T} (k)\hat{\theta }(k - 1)} \right]} \\ {K(k) = \frac{{P(k - 1)\theta (k)}}{{\lambda + \varphi ^{T} (k)P(k - 1)\varphi (k)}}} \\ {P(k) = \frac{1}{\lambda }\left[ {I - K(k)\varphi ^{T} (k)} \right]P(k - 1)} \\ \end{array} } \right.$$

### Summary

After the parameters of the model are identified, we can use the state estimation method to predict the core temperature of the battery according to the thermal model. At present, the joint Kalman filter is a good and often used method. However, there are still shortcomings in this method. Because the two-node equivalent thermal network model of the battery has been greatly simplified, there is a certain error between the estimation of the core temperature and the real value. The two-node equivalent thermal network model assumes that some parameters are constant, but in fact, due to the changes of battery temperature and SOC, the parameters of the model are usually time-varying. The identification of time-varying parameters is time-consuming work. So this paper designs a multi-step ahead thermal warning network for the energy storage system based on the core temperature detection.

## Methods

### Model

#### Multi-step ahead thermal warning network

**Figure 3:**
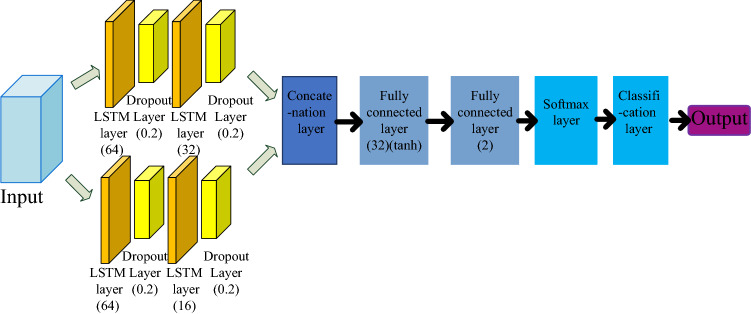
Multi-step ahead thermal warning network model.

The multi-step ahead thermal warning network established in this paper is shown in Fig. 3*.* The number of neurons in each hidden layer and the activation function used are illustrated in brackets in Fig. 3. The input of the model connects two integrated LSTM networks, each of which includes two LSTM layers. The number of neurons in the two LSTM layers of the first LSTM network is 64 and 32 respectively, and the number of neurons in the second LSTM network is 64 and 16 respectively. A dropout layer is added after each LSTM layer to prevent overfitting. The probability of dropout is 0.5. Then, the outputs of two LSTM networks are connected in series through the concatenation layer. Then two fully connected layers are connected. The number of neurons in the first fully connected layer is 32, and the activation function is tanh. The number of neurons in the second fully connected layer is 2. The second fully connected layer determines the number of categories. Finally, the output is obtained through the softmax layer and the classification layer, and the classification layer takes the cross entropy as the loss function. The LSTM cell, dropout layer, softmax and cross entropy are explained in detail in the next section.

**Figure 4:**
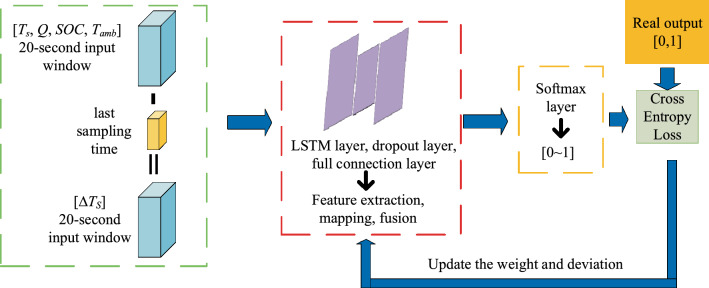
The workflow of multi-step ahead thermal warning network.

The workflow and schematic diagram of the multi-step ahead thermal warning network for the energy storage system is shown in Fig. 4. The sampling time Δt set in this paper is 1 s. The inputs of the neural network are the measured surface temperature *T*_*s*_, heat *Q*, SOC of lithium battery, ambient temperature *T*_*amb*,_ and the surface temperature difference Δ*T*_*s*_ between the two adjacent sampling times for 20 s. Then the inputs are passed to the hidden layer. Features are extracted and mapped through the integrated LSTM layer, and fused through the fully connected layer. Then the output of the fully connected layer refracts to the probability between 0–1 through the softmax layer. The real output is 0 and 1. 0 means that the core temperature of the lithium battery energy storage system will not reach the critical value in the next 10 s, and the warning should not be given; 1 means that the core temperature may exceed the limit in the next 10 s, and the energy storage system operation may be dangerous, which needs early warning. Then the output of softmax and the real output calculate the network loss through cross entropy, and calculate the gradient of loss to update the weight and deviation of each hidden layer. This process is repeated until the end of the iteration.

### Network layer interpretation

#### LSTM cell

**Figure 5:**
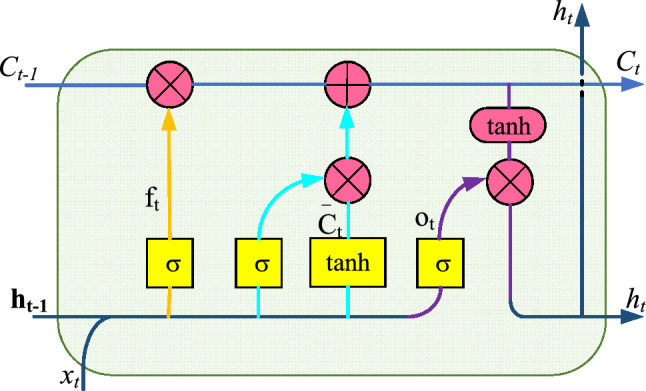
Structure of long short-term memory cell.

LSTM network is a recurrent neural network (RNN). RNN network is a kind of artificial neural network for sequence data; it attempts to simulate time-related or sequence-related behavior^31^. The hidden structure of RNN is the memory of the network, and the current state is affected by both the current input and the previous state. This structure enables RNN to effectively process time series data^32^. With the development of back-propagation and computing efficiency, RNN has been applied in various fields. However, RNN performs poorly in long-term sequences, and information from earlier moments has little effect on the output of the current moment. Another disadvantage of RNN is that gradient disappearance and gradient explosion may occur during back-propagation. This is because small gradients or weights multiply many times in multiple time intervals, and gradients will shrink to zero asymptotically. However, when the gradient is too small or disappears, the network can’t adjust the weight in the direction of reducing the error, which makes the RNN network stop learning and cannot learn the long-term dependence^33^. In order to overcome the long-term dependence of RNN, Jürgen Schmidhuber et al. proposed a recursive neural network with long and short-term memory in 1997^34^. Compared with simple RNN, LSTM adds a state in the hidden layer to maintain the long-term state, and this newly added state is called cell state^35^. The input of LSTM includes the output value, the input value and the cell state of the previous time, and the output includes the output value and the cell state of the current time. Similar to the classical RNN, LSTM is composed of input layer, hidden layer and output layer. And LSTM has the form of repetitive module chain of neural network. However, the hidden layer of LSTM adopts a special memory mechanism, and the repeat module of LSTM has different structures. References^36–41^ explained the working mechanism of the LSTM unit. The structure of the LSTM cell is shown in Fig. 5.

The top line from *C*_*t*−1_ to *C*_*t*_ in Fig. 2 represents the transport of cell state, which is also the key to LSTM. LSTM acquires the ability to delete or add information to the cell state through a structure called gate. The gate consists of a Sigmod neural network layer and a bitwise multiplication operation. The activation function of the Sigmod neural network layer is shown in Eq. (11). It can convert the input signal to a value between 0 and 1, so as to determine how many input signals can pass through. 0 means no signal is allowed to pass, 1 means all signals are allowed to pass.

11 $$\sigma (x) = \frac{1}{{1 + {\text{e}}^{{ - x}} }}$$

LSTM consists of three kinds of gates: forgetting gate, input gate and output gate^42^.

The forgetting gate determines what information should be forgotten in the previous cell state *C*_*t*−1_. First, the output *h*_*t*−1_ of the previous time and the input *x*_*t*_ of the current time are accepted, and the signal *f*_*t*_ is output through the Sigmod layer. Then *f*_*t*_ is a value from 0 to 1, which is multiplied by *C*_*t*−1_ to determine the information retained in *C*_*t*−1_. As shown in Eq. (12).

12 $$f_{t} = \sigma (W_{f} \cdot [h_{{t - 1}} ,\;x_{t} ] + b_{f} )$$

The input gate determines the new information to be entered in the cell state. First, *x*_*t*_ and *h*_*t*−1_ are input into the Sigmod layer, and a value *i*_*t*_ between 0 and 1 is output. At the same time, *x*_*t*_ and *h*_*t*−1_ create a new state candidate vector $\overline{C} _{t}$ with values between − 1 and 1 through a tanh neural network layer. Then *i*_*t*_ and $\overline{C} _{t}$ are multiplied to determine which information is added to the cell state $\overline{C} _{t}$ at the current time. As shown in Eqs. (13) and (14).

13 $$i_{t} = \sigma (W_{i} \cdot [h_{{t - 1}} ,\;x_{t} ] + b_{i} )$$

14 $$\bar{C}_{t} = \tanh (W_{c} \cdot [h_{{t - 1}} ,\;x_{t} ] + b_{c} )$$

The cell state is updated according to the output of the forgetting gate and the input gate, as shown in Eq. (15).

15 $$C_{t} = f_{t} \times C_{{t - 1}} + i_{t} \times \bar{C}_{t}$$

The output gate determines what the LSTM cell outputs. First, *x*_*t*_ and *h*_*t*−1_ are input into Sigmod layer, and a value *o*_*t*_ between 0 and 1 is output. Then the updated cell state *Ct* is converted to a function between − 1 and 1 by a tanh function (as shown in Eq. (16)). The new output is obtained by multiplying *o*_*t*_ and *Ct*, which is also the input signal at the next moment. The process is shown in Eqs. (17) and (18).

16 $$\tanh (x) = \frac{{e^{x} - e^{{ - x}} }}{{e^{x} + e^{{ - x}} }}$$

17 $$o_{t} = \sigma (W_{o} [h_{{t - 1}} ,\;x_{t} + b_{o} ])$$

18 $$h_{t} = o_{t} *\tanh (C_{t} )$$

#### Dropout

**Figure 6:**
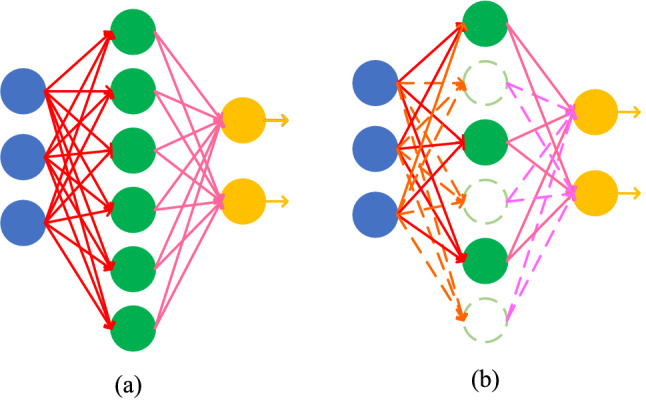
The principle of dropout: (**a**) real network layer; (**b**) network layer through dropout.

Dropout layer is to prevent overfitting due to many model parameters but few training samples^43^. The principle of Dropout is shown in Fig. 6.

Firstly, a certain proportion of hidden neurons are randomly deleted, and the input and output neurons remain unchanged. Then the input is propagated forward through the modified network, and the loss is propagated back through the modified network, so that only the neurons that have not been deleted will update the corresponding parameters. Repeat the process. In this way, every two hidden nodes may not appear at the same time, and the update of weights no longer depends on the interaction of hidden nodes with fixed relationship, which prevents some features from having effect only under other specific features. In addition, Dropout can also be regarded as an average model. Hidden neurons are deleted randomly each time, so different samples correspond to different models.

#### Softmax and cross entropy

In deep learning, Softmax and Cross entropy are common and important functions. And Softmax and Cross entropy are usually used in multi-classification neural networks.

Suppose that the original outputs of the neural network are y_1_, y_2_,…, y_n_. Then the output after Softmax is shown in Eq. (19).

19 $${\text{softmax}}\;(y_{i} ) = \frac{{e^{{y_{i} }} }}{{\mathop \sum \nolimits_{{j = 1}}^{n} e^{{y_{i} }} }}$$

So Softmax can map the input to a real number between 0 and 1, and ensure that the sum is 1, so that the output of each node becomes a probability value.

Cross entropy is usually used as the loss function of the network to determine the closeness between the actual output and the expected output. As shown in Eq. (20), the probability distribution *p* is the expected output and the probability distribution *q* is the actual output. Cross entropy represents the distance between the actual output probability and the expected output probability. The smaller the cross entropy is, the closer the two probability distributions are.

20 $$H(p,\;q) = - \mathop \sum \limits_{i} p_{i} logq_{i}$$

The combination of Softmax function and Cross entropy function can greatly simplify the gradient of loss function. The formula is shown in Eq. (21). Where *L* is the cross-entropy loss function; *b*_*i*_ is the real distribution; *a*_*i*_ is the predicted distribution, that is, the output of Softmax; and *z*_*i*_ is the output of neurons.

$$L = - \mathop \sum \limits_{i} b_{i} lna_{i}$$

21 $$a_{i} = {\text{softmax}}\;(z_{i} )$$

$$\frac{{\partial L}}{{\partial z_{i} }} = a_{i} - b_{i}$$

## Results

### Parameter setting and training network

**Figure 7:**
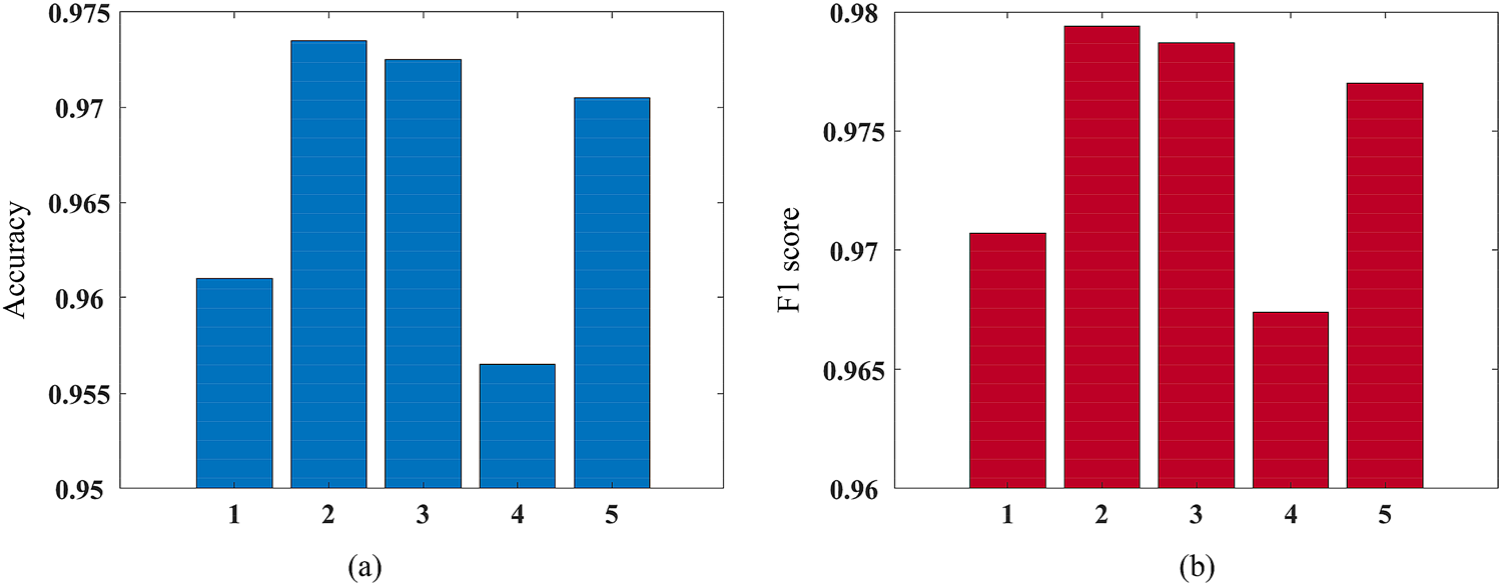
The accuracy and F1 score of networks with different inputs: (**a**) accuracy; (**b**) F1 score. Where 1 represents the network input as [T_s_, Q, SOC, T_amb_], 2 represents input as [T_s_, Q, SOC, T_amb_. ΔT_s_], 3 represents input as [T_s_, Q, SOC, T_amb_, ΔT_s_, ΔQ], 4 represents input as [T_s_, Q, SOC, T_amb_, ΔT_s_, ΔSOC], and 5 represents input as [T_s_, Q, SOC, T_amb_, ΔT_s_, ΔQ, ΔSOC].

**Figure 8:**
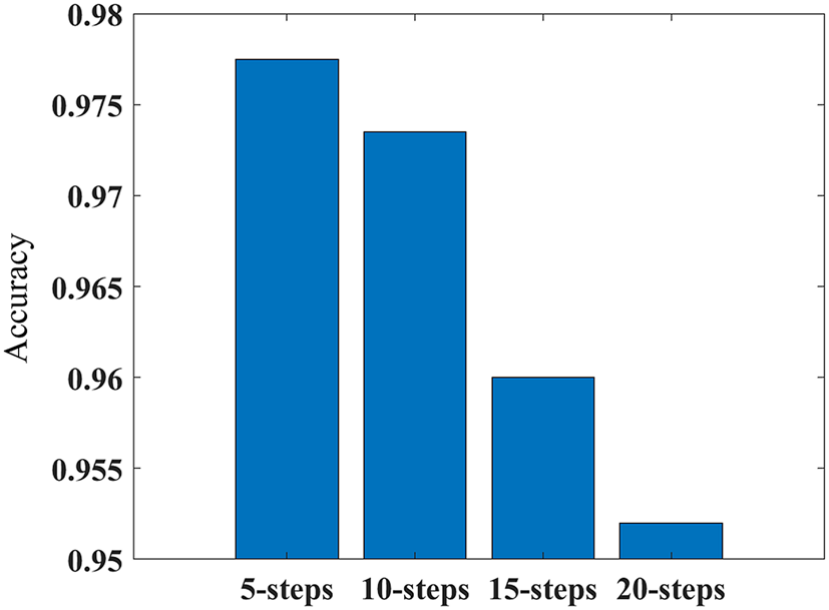
The accuracy of networks with different predicted time-steps.

**Figure 9:**
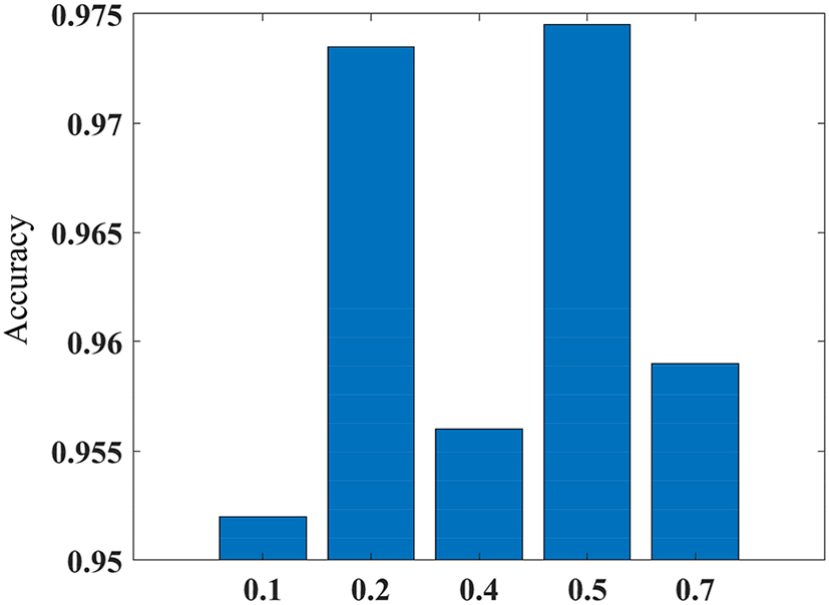
The accuracy of networks with different dropout parameters.

**Figure 10:**
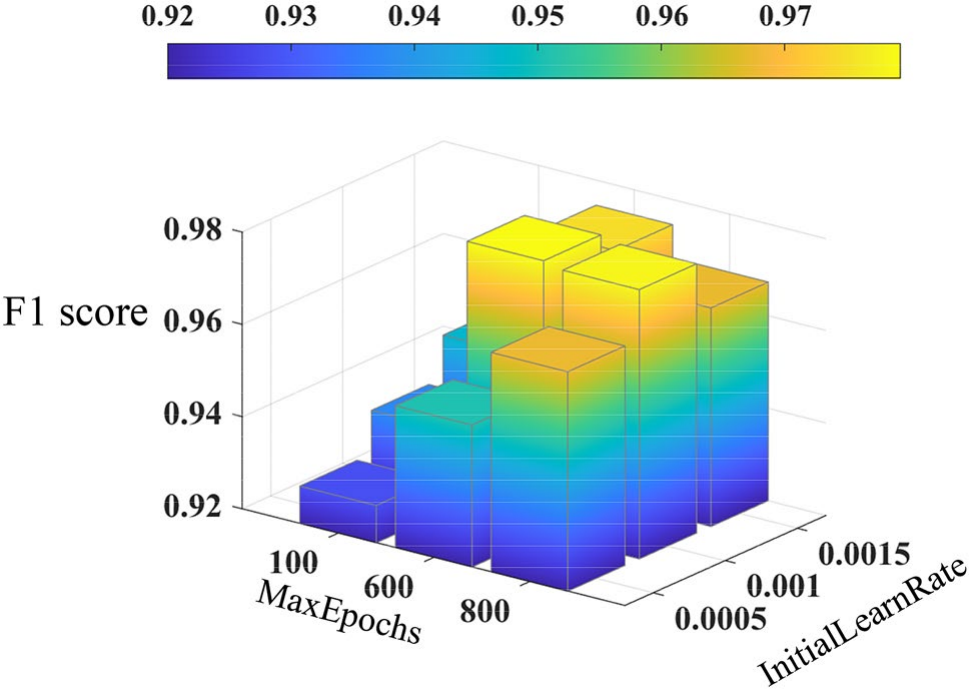
The F1 score of networks with different Maxepochs and InitialLearnrate.

This paper uses MATLAB to establish the multi-step ahead thermal warning network and training network. In this paper, “Adam” algorithm is used. This paper changes the network input, predicted time-steps of the network output, training parameters and the probability of dropout. Then we test different networks. The results of the tests are shown in Figs. 7, 8, 9 and 10. In Fig. 7, We can see that when the difference between two sampling points is added to the model input, the accuracy and F1 score of the model are improved. When the temperature difference and heating difference are added to the model input, the effect of the model can be greatly improved. However, the accuracy and F1 score of the model will be decreased by adding the SOC difference. The results in Fig. 8 show that the accuracy of the model will decrease with the increase of the model’s predicted time steps. When the number of time steps is five or ten, the prediction accuracy is higher. And when the number of time steps is further increased, the accuracy of the model decreases greatly. Figure 9 shows that when the dropout parameter is small, the ability to prevent overfitting is poor, and the accuracy of the model is also low. When the dropout parameter is too large, the accuracy of the model will also decline. 0.5 should be a good value for the dropout parameter, which can make the accuracy of the model reach 97%. In Fig. 10, we can see that when the training epochs are small or the initial learning rate is small, increasing the other side can effectively improve the prediction ability of the model. However, when both of them are large, the model may be overtrained and the generalization ability of the model will be reduced.

**Figure 11:**
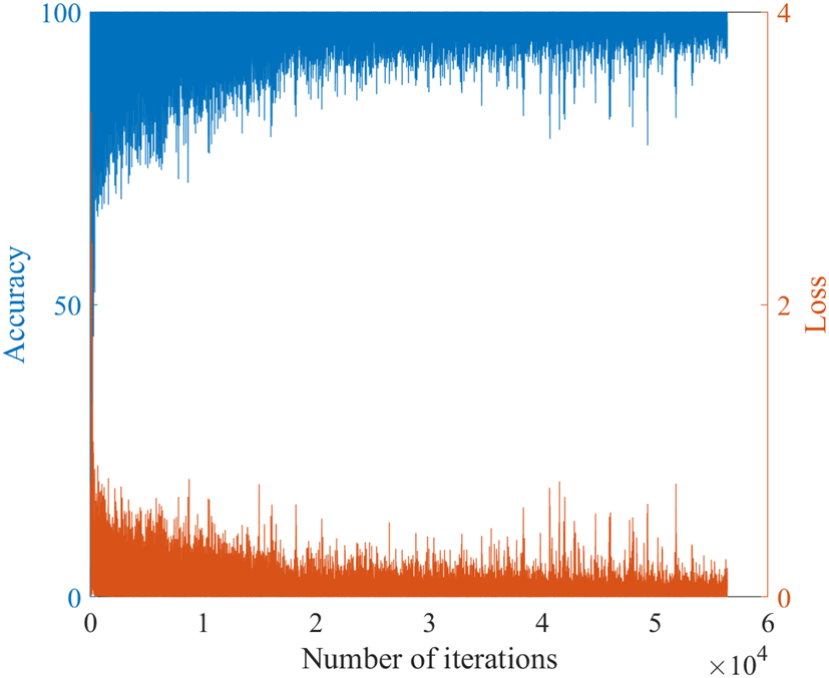
The training-process of the multi-step ahead thermal warning network.

The best inputs are the surface temperature *T*_*s*_, heat *Q*, SOC of lithium battery, ambient temperature *T*_*amb*_, and the surface temperature difference Δ*T*_*s*_. The probability of dropout is 0.5. The best training parameters are set as follows. ‘ExecutionEnvironment’ is cpu; ‘MaxEpochs’ is 600; ‘SequenceLength’ is longest; ‘GradientThreshold’ is 1; ‘InitialLearnRate’ is 0.001. In this paper, 12,100 s lithium battery data is used to generate the training input of the neural network model by the sliding window method. And 0–1 value is generated according to whether the core temperature of the lithium battery in the next 10 s corresponding to each window reaches the critical value, which is regarded as the training output of the neural network model. The length of each sliding window is 20 s. After automatic training, a better multi-step ahead thermal warning network model is obtained. The training-process curve is shown in Fig. 11.

### Accuracy

Accuracy is the ratio of the number of samples correctly classified to the total number of samples. It is shown in Eq. (22).

22 $$accuracy = \frac{{TP + TN}}{{TP + TN + FP + FN}} = 0.9750$$

TP represents the number of samples with the real value of 1 and the predicted value of 1.

TN represents the number of samples with the real value of 0 and the predicted value of 0.

FP represents the number of samples with the real value of 0 and the predicted value of 1.

FN represents the number of samples with the real value of 1 and the predicted value of 0.

### Precision

Precision represents the proportion of the number of correct predictions in the result with a prediction of 1. Its significance is to judge whether the results of the model can "find the right one". It is shown in Eq. (23).

23 $$precision = \frac{{TP}}{{TP + FP}} = 0.9794$$

### Recall

Recall represents the proportion of the number of samples correctly predicted in samples with a real value of 1. Its significance is to judge whether the results of the model can “find all”. As shown in Eq. (24).

24 $$recall = \frac{{TP}}{{TP + FN}} = 0.9824$$

### F1-score

F1 score is the harmonic average of precision and recall. This is because precision and recall are sometimes contradictory indicators, and they need to be considered together. As shown in Eq. (25).

25 $$F1 = \frac{{2 \cdot precision \cdot recall}}{{precision + recall}} = 0.9809$$

## Conclusion

In this paper, a novel multi-step ahead thermal warning network is proposed for the energy storage system as the core temperature overrun warning. Various methods are compared to prove the accuracy advantage of the proposed model. By changing the input of the model, it can be seen that when only adding the surface temperature difference of two sampling points, the accuracy and F1 score of the model are the highest and can reach more than 97%. Besides, the insertion of the dropout layer to the thermal warning network benefits the predicted effect of the model. When the dropout parameters are selected properly, the accuracy of the model can be improved by about 2%. During the process of network training, the integrated selection of MaxEpochs and InitialLearnRate has a great influence on the model. Both overtraining and undertraining of the network will reduce the generalization capability of the network. The highest accuracy of the proposed multi-step ahead thermal warning network can reach more than 97%. It can be found that the prediction step of 10-steps is also effective. The proposed network can accurately estimate the core temperature in the next 10 s and provide the early-warning signal according to the measurement of the previous 20 s, which saves significant time for the thermal protection of the large-scale energy storage system.

## Data Availability

The datasets generated during the current study are available from the corresponding author on reasonable request.

## References

[1] Amini M, Karabasoglu O (2018). Optimal operation of interdependent power systems and electrified transportation networks. Energies.

[2] Fan B (2018). An adaptive neuro-fuzzy inference system (ANFIS) based model for the temperature prediction of lithium-ion power batteries. SAE Int. J. Passeng. Cars Electron. Electr. Syst..

[3] Kim J, Oh J, Lee H (2019). Review on battery thermal management system for electric vehicles. Appl. Therm. Eng..

[4] Arora P, White RE, Doyle M (1998). Capacity fade mechanisms and side reactions in lithium-ion batteries. J. Electrochem. Soc..

[5] Alipour M, Esen E, Kizilel R (2019). Investigation of 3-D multilayer approach in predicting the thermal behavior of 20 Ah Li-ion cells. Appl. Therm. Eng..

[6] Richardson RR, Ireland PT, Howey DA (2014). Battery internal temperature estimation by combined impedance and surface temperature measurement. J. Power Sources.

[7] Kim, H., Kim, S., Kim, T., Hu, C. & Youn, B. D. Online thermal state estimation of high power lithium-ion battery. In *2015 IEEE Conference on Prognostics and Health Management *(*PHM*) 1–6 (IEEE, 2015). 10.1109/ICPHM.2015.7245067.

[8] Zhang G (2014). In situ measurement of radial temperature distributions in cylindrical Li-Ion cells. J. Electrochem. Soc..

[9] Gümüşsu E, Ekici Ö, Köksal M (2017). 3-D CFD modeling and experimental testing of thermal behavior of a Li-Ion battery. Appl. Therm. Eng..

[10] Kanbur BB, Kumtepeli V, Duan F (2020). Thermal performance prediction of the battery surface via dynamic mode decomposition. Energy.

[11] Xie Y (2020). A novel electro-thermal coupled model of lithium-ion pouch battery covering heat generation distribution and tab thermal behaviours. Int. J. Energy Res..

[12] Xie Y (2020). An improved resistance-based thermal model for prismatic lithium-ion battery charging. Appl. Therm. Eng..

[13] Wang, D., Gao, Y., Zhang, X., Dong, T. & Zhu, C. A novel pseudo two-dimensional model for NCM Liion battery based on electrochemical-thermal coupling analysis. In *2020 3rd International Conference on Electron Device and Mechanical Engineering *(*ICEDME*) 110–116 (IEEE, 2020). 10.1109/ICEDME50972.2020.00031.

[14] Cui X (2020). Simplification strategy research on hard-cased Li-ion battery for thermal modeling. Int J Energy Res.

[15] Saqli, K., Bouchareb, H., M’sirdi, K. N., Naamane, A. & Oudghiri, M. Electric and thermal model of Li-ion battery pack with cylindrical components. In *2020 5th International Conference on Renewable Energies for Developing Countries *(*REDEC*) 1–6 (IEEE, 2020). 10.1109/REDEC49234.2020.9163865.

[16] Chen L (2020). Core temperature estimation based on electro-thermal model of lithium-ion batteries. Int. J. Energy Res..

[17] Zhang C, Li K, Deng J (2016). Real-time estimation of battery internal temperature based on a simplified thermoelectric model. J. Power Sources.

[18] Ruan H, Jiang J, Ju Q, Sun B, Cheng G (2017). A reduced wide-temperature-range electro-thermal model and thermal parameters determination for lithium-ion batteries. Energy Procedia.

[19] Barcellona S, Piegari L (2017). Lithium ion battery models and parameter identification techniques. Energies.

[20] Anthony D, Sarkar D, Jain A (2016). Non-invasive, transient determination of the core temperature of a heat-generating solid body. Sci. Rep..

[21] Anthony D, Wong D, Wetz D, Jain A (2017). Non-invasive measurement of internal temperature of a cylindrical Li-ion cell during high-rate discharge. Int. J. Heat Mass Transf..

[22] Chun H, Kim J, Yu J, Han S (2020). Real-time parameter estimation of an electrochemical lithium-ion battery model using a long short-term memory network. IEEE Access.

[23] Li D, Zhang Z, Liu P, Wang Z, Zhang L (2021). Battery fault diagnosis for electric vehicles based on voltage abnormality by combining the long short-term memory neural network and the equivalent circuit model. IEEE Trans. Power Electron..

[24] Wang, X., Zhao, T., Liu, H. & He, R. Power consumption predicting and anomaly detection based on long short-term memory neural network. In *2019 IEEE 4th International Conference on Cloud Computing and Big Data Analysis *(*ICCCBDA*) 487–491 (IEEE, 2019). 10.1109/ICCCBDA.2019.8725704.

[25] Feng F (2020). Co-estimation of lithium-ion battery state of charge and state of temperature based on a hybrid electrochemical-thermal-neural-network model. J. Power Sources.

[26] Tang X, Yao K, Liu B, Hu W, Gao F (2018). Long-term battery voltage, power, and surface temperature prediction using a model-based extreme learning machine. Energies.

[27] Park C, Jaura AK (2003). Dynamic thermal model of li-ion battery for predictive behavior in hybrid and fuel cell vehicles. SAE Tech. Pap..

[28] Perez, H. E. *et al.* Parameterization and validation of an integrated electro-thermal cylindrical LFP battery model. In *DSCC2012-MOVIC2012* 41–50 (2012). 10.1115/DSCC2012-MOVIC2012-8782.

[29] Forgez C, Vinh Do D, Friedrich G, Morcrette M, Delacourt C (2010). Thermal modeling of a cylindrical LiFePO_4_/graphite lithium-ion battery. J. Power Sources.

[30] Lin X (2013). Online parameterization of lumped thermal dynamics in cylindrical lithium ion batteries for core temperature estimation and health monitoring. IEEE Trans. Control Syst. Technol..

[31] Hong J, Wang Z, Yao Y (2019). Fault prognosis of battery system based on accurate voltage abnormity prognosis using long short-term memory neural networks. Appl. Energy.

[32] Li, C., Xiao, F., Fan, Y., Yang, G. & Zhang, W. A recurrent neural network with long short-term memory for state of charge estimation of lithium-ion batteries. In *2019 IEEE 8th Joint International Information Technology and Artificial Intelligence Conference *(*ITAIC*) 1712–1716 (IEEE, 2019). 10.1109/ITAIC.2019.8785770.

[33] Hong J, Wang Z, Chen W, Yao Y (2019). Synchronous multi-parameter prediction of battery systems on electric vehicles using long short-term memory networks. Appl. Energy.

[34] Hochreiter S, Schmidhuber J (1997). Long short-term memory. Neural Comput..

[35] Chen Z (2020). Capacity prediction and validation of lithium-ion batteries based on long short-term memory recurrent neural network. IEEE Access.

[36] Li W (2021). Online capacity estimation of lithium-ion batteries with deep long short-term memory networks. J. Power Sources.

[37] Shin D, Yoon B, Yoo S (2021). Compensation method for estimating the state of charge of li-polymer batteries using multiple long short-term memory networks based on the extended kalman filter. Energies.

[38] Tan Y, Zhao G (2020). Transfer Learning With long short-term memory network for state-of-health prediction of lithium-ion batteries. IEEE Trans. Ind. Electron..

[39] Fasahat M, Manthouri M (2020). State of charge estimation of lithium-ion batteries using hybrid autoencoder and long short term memory neural networks. J. Power Sources.

[40] Chang F, Chen T, Su W, Alsafasfeh Q (2020). Control of battery charging based on reinforcement learning and long short-term memory networks. Comput. Electr. Eng..

[41] Tian Y, Lai R, Li X, Xiang L, Tian J (2020). A combined method for state-of-charge estimation for lithium-ion batteries using a long short-term memory network and an adaptive cubature Kalman filter. Appl. Energy.

[42] Dong C, Chu R, Morstyn T, McCulloch MD, Jia H (2021). Online rolling evolutionary decoder-dispatch framework for the secondary frequency regulation of time-varying electrical-grid-electric-vehicle system. IEEE Trans. Smart Grid.

[43] Srivastava N, Hinton G, Krizhevsky A, Sutskever I, Salakhutdinov R (2014). Dropout: A simple way to prevent neural networks from overfitting. J. Mach. Learn. Res..

